# A combinatorial approach for detecting gene-gene interaction using multiple traits of Genetic Analysis Workshop 16 rheumatoid arthritis data

**DOI:** 10.1186/1753-6561-3-s7-s43

**Published:** 2009-12-15

**Authors:** Xiaoqi Cui, Qiuying Sha, Shuanglin Zhang, Huann-Sheng Chen

**Affiliations:** 1 Department of Mathematical Sciences, Michigan Technological University, 1400 Townsend Drive, Houghton, Michigan 49931, USA

## Abstract

Rheumatoid arthritis is inherited in a complex manner. So far several single susceptibility genes, such as *PTPN22*, *STAT4*, and *TRAF1-C5*, have been identified. However, it is presumed that some genes may interact to have a significant effect on the disease, while each of them only plays a modest role. We propose a new combinatorial association test to detect the gene-gene interaction in the rheumatoid arthritis data using multiple traits: disease status, anti-cyclic citrullinated peptide, and immunoglobulin M. Existing gene-gene interaction tests only use the information on a single trait at a time. In this article, we propose a new multivariate combinatorial searching method that utilizes multiple traits at the same time. Multivariate combinatorial searching method is conducted by incorporating the multiple traits with various techniques of feature selection to search for a set of disease-susceptibility genes that may interact. By analyzing three panels of markers, we have identified a significant gene-gene interaction between *PTPN22 *and *TRAF1-C5*.

## Background

Rheumatoid arthritis (RA) has a complex mode of inheritance that involves the interaction of several genes and environmental factors. So far, several genes have been identified as conferring high to modest level of risk to RA, including *PTPN22 *[1], *STAT4 *[2], and *TRAF1-C5 *[3]. Due to computational complexity, most association studies evaluate one locus or gene at a time. However, there is evidence suggesting that gene-gene interaction may play an important role in complex disease [4]. To detect genes that may interact, some methods have been proposed [5,6], but they only work on a single trait at a time. For a complex disease, the data is often collected with information on several traits. Analyzing the association using multiple traits jointly with the genotypes may have advantage in improving the power of the test. Inspired by the combinatorial partitioning [5] and searching [6] methods, we propose a new combinatorial association test incorporating multiple traits to detect gene-gene interaction in the RA data in Genetic Analysis Workshop 16 (GAW16) Problem 1. Unlike the methods of Nelson et al. [5] and Sha et al. [6], our test utilizes the information on multiple traits (disease status, anti-cyclic citrullinated peptide (anti-CCP) and immunoglobulin M (IgM)) at the same time. Moreover, to improve computational efficiency incurred by incorporating multiple traits, we use the *K*-mean clustering method to reduce the dimension, as well as the Akaike's information criterion (AIC) to select models.

## Methods

### Marker selection

We selected 151 single-nucleotide polymorphisms (SNPs) from the three genes which are mentioned above. For *PTPN22*, 41 consecutive SNPs (from rs10858004 to rs11485101) in and around the *PTPN22 *gene were included. The recently reported SNP rs2476601 (encoding R620W) is the center of the 41 markers that span an interval of more than 400 kb. rs2476601 is well known to be associated with multiple autoimmune diseases, including RA [1].

For *STAT4*, we selected 51 SNPs (from rs883844 to rs6744978) in *STAT1-STAT4 *region [2] covering more than 200 kb. Remmers et al. [2] conducted case-control analysis and located rs7574865 as the most significant SNP associated with disease in this region.

In the *TRAF1-C5 *region on chromosome 9, we tested 59 SNPs (from rs10739575 to rs4837820) that span a region of about 400 kb covering all the SNPs genotyped by Plenge et al. [3] and Kurreeman et al. [7]. Plenge et al. [3] located the most significant SNP rs3761847, and Kurreeman et al. [7] also confirmed the importance of the *TRAF1-C5 *region.

### Data cleaning

We removed the SNPs that have only one type of genotype or with genotype missing rate ≥ 5%. After genotype imputation (see below "Genotype and phenotype imputation"), the data were further filtered based on minor allele frequency (MAF) < 5% and the *p*-value of HWE test <0.001. Finally, 137 SNPs remained.

We also discarded the diseased individuals with missing anti-CCP or IgM measures. To minimize the effect of outliers, we removed individuals with anti-CCP ≥ 500 or IgM ≥ 800. After the above data trimming, 1860 individuals (1194 controls and 666 cases) remained for analysis.

### Genotype and phenotype imputation

Missing genotypes for each SNP were randomly imputed based on the marginal distribution of the genotypes in all other individuals.

**Figure 1:**
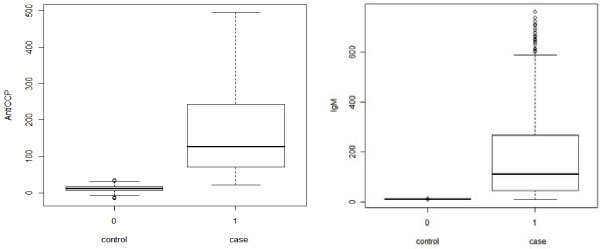
**Distributions of anti-CCP and IgM in cases and controls after phenotype imputation**.

Because all anti-CCP and IgM values are missing in controls, we imputed these two values for each control from a bivariate normal distribution whose means (11.1 for anti-CCP and 12 for IgM) and variances (58.5225 and 0.4) are taken from the existing literatures [8,9]. The correlation (0.074) between anti-CCP and IgM in controls is assumed to be the same as in the 666 trimmed cases. After imputation, the distributions of anti-CCP and IgM in cases and controls were compared by boxplots (Figure 1). We found that the imputed trait values successfully distinguish the cases and controls: there is a large difference in trait values between cases and controls, and even the maximum trait values in controls are below the traits' first quartile in cases. Moreover, after imputation, the general correlation between anti-CCP and IgM (combining cases and controls) is 0.117, a little bit larger than 0.074, which is expected.

### The MCSM algorithm

The goal of the multivariate combinatorial searching method (MCSM) is to identify a set of loci that are associated with the multiple traits. Suppose that, within one or several genes, *K *SNP loci are genotyped. Consider the subsets of the *K *SNPs, there are  hoosing *L *from *K*) different locus-sets that involve *L *SNPs (1 ≤ *L *≤ *K*). *L *is a pre-specified number, and in our implementation, we only consider single-locus sets (*L *= 1) and two-locus sets (*L= *2). For the *N *individuals, the *i*^th ^individual has a trait vector ***y***_*i *_= (***y***_*i*1_, ***y***_*i*2_, ***y***_*i*3_)^*T*^, ***i ***= 1, ..., ***N***. Let . The procedure of MCSM can be divided into three steps:

#### Step 1. Filter all possible L-locus sets

In Step 1, we search all *L*-locus sets (*L *= 1,2) and use a filter to retain those sets that can explain a significant proportion of the total variation in the multiple traits. For a *L*-locus set (*L *= 1, 2) let *g*_1_, ..., *g*_*m*+1 _denote all the distinct multi-locus genotypes observed in the sample, where *m*+1 is the total number of distinct multi-locus genotypes in this sample. For example, if *L *= 1, then *m *= 2. The *i*^th ^individual has corresponding genotypic coding as *X*_*i *_= (*x*_*i*1_, ..., *x*_*im*_) where

Consider a multivariate linear regression model:

where the coefficient *β*_*j *_= (*β*_*j*1_, *β*_*j*2_, *β*_*j*3_)^*T*^, *j *= 1, ..., *m*, and *ε*_*I *_= (*ε*_*i*1_, *ε*_*i*2_, *ε*_*i*3_)^*T*^, *i *= 1, ..., *N *is a three-dimensional random vector, independent of the genotypic score *X*_*i*_

Equivalently, Eq. (1) can be rewritten as

where *Y *is denoted as above, *X *= (*J*, *X*_1_, *X*_2_, *...*, *X*_*N*_)^*T *^and *J *is a vector whose elements are 1's, *B *= (*β*_0_, *β*_1_, ..., *β*_*m*_)^*T *^and ℑ = (*ε*_1_, *ε*_2_, ..., *ε*_*N*_)^*T*^.

Based on the multivariate model in Eqs. (1) or (2), a natural choice of filter for screening out the insignificant SNP loci is an *R*^2^-like measure of association between *Y *and *X *:  where *S*_*yy *_is the sample covariance matrix of the *y *values, *S*_*xx *_is the sample covariance matrix of *x *values, and *S*_*xy *_is the matrix of sample covariance between *y *values and *x *values. However,  is too small to meaningfully reflect the amount of association. Therefore, we use *r*, the square root of the maximum eigenvalue of , which is also the largest canonical correlation between *Y *and *X*. Within all *L*-locus sets, after calculating the *r *value for each set, we retain the top 10 sets that have the largest *r *values for future validation (step 2).

#### Step 2. Validate and compare the locus-sets retained in Step 1

In Step 2, we validate and compare those locus sets retained in Step 1. Because *m *becomes large rapidly as *L *increases, *K*-mean clustering method is used to reduce the high dimension [6]. Then for a two-locus set (nine genotypes), we only need evaluate eight (k = 2,3,...,9) different partitions of the nine genotypes, instead of 21,146 [5]. After partitioning the genotypes into *k *groups, denote G_1_,...,G_*k *_to be the *k *genotype groups found by the *K*-mean clustering method and then redefine a numerical code for the genotype of the *i*^th ^individual as *X*_*i *_= (*x*_*i*,1, ..., _*x*_*i*, *k*-1_), where

In this way, we construct a new multivariate regression model for each retained locus set, which is similar to Eqs. (1) or (2). The AIC of the multivariate model is a measure that indicates the reliability of the model. The small value of AIC means that the model or the locus set is reliable. For a specific locus set, the AIC value for each partition is calculated, and the smallest AIC is chosen as the final value of the objective function of that locus set:

At the end of Step 2, the best locus set is chosen as the one that has the smallest value of the objective function *T*.

#### Step 3. Assess the statistical significance

To assess the statistical significance of the best locus set chosen from Step 2, we perform a permutation test. Denote the objective function (*T*) value of the best locus set as *T*_0_. In each permutation, the trait vectors *y *= (*y*_1_, *y*_2_, *y*_3_)^*T *^of the individuals are randomly permuted, then Step 1 and 2 are repeated on the randomized data. A objective function (*T*) value is obtained for each permutation. For 1000 permutations, denote values of the objective function (*T*) by *T*_1_, *T*_2_, ..., *T*_1000_. The overall *p*-value of the test for association between the final locus set and the multiple trait is 

### Data simulation

Before applying the MCSM to the GAW 16 data, the method was evaluated through simulated data sets. To assess the type I error, we generated genotypes at ten independent markers by the MAF (assuming equal MAF across the ten markers); the phenotype data is the same as the one used in GAW16. To evaluate the power of MCSM, we generated both genotype and phenotype data based on the dichotomous trait in GAW16. Here we simulated the checkerboard model [6] with two functional SNPs. From the dichotomous trait in GAW16 data (666 cases and 1194 controls), the genotypes at two functional SNPs were generated. Then according to the genotypes at the two functional loci, the two quantitative traits can be generated [6]. For testing both type I error and power, 100 replicates with 100 permutations were conducted, and MAF varied from 0.1 to 0.5.

## Results

### Simulated data

When the level of significance is 0.05, the type I errors of the MCSM are 0.05 for an MAF of 0.1 and 0.5, respectively, and power was 100% for both MAF of 0.1 and 0.5. Thus, when combining multiple traits, the power of the MCSM is very high.

### GAW16 RA data

After implementing MCSM on 1860 individuals with 137 SNPs, we located the best set as a two-locus set: rs7037673 (*TRAF1-C5*) and rs2476601 (*PTPN22*) with a *p*-value of 0.031. Table 1 lists the top five two-locus sets and Table 2 lists the top five single-locus sets as well as their *p*-values. As we can see, no single-locus set is significant, and even the *T *value of the best single-locus set is still greater (worse) than the *T *values of all retained two-locus sets, which could be evidence of the dominance of joint (gene-gene interaction) effects upon marginal effects of the candidate genes.

**Table 1:** Top five two-locus sets

SNP 1 (gene)	Chr	SNP 2 (gene)	Chr	***p*-value**
rs7037673 (*TRAF1-C5*)	9	rs2476601 (*PTPN22*)	1	0.031
rs17611 (*TRAF1-C5*)	9	rs2476601 (*PTPN22*)	1	0.058
rs3761847 (*TRAF1-C5*)	9	rs2476601 (*PTPN22*)	1	0.067
rs993247 (*TRAF1-C5*)	9	rs2476601 (*PTPN22*)	1	0.08
rs1951784 (*TRAF1-C5*)	9	rs2476601 (*PTPN22*)	1	0.093

**Table 2:** Top five single-locus sets

SNP (gene)	Chr	*p*-value
rs2476601 (*PTPN22*)	1	0.942
rs3761847 (*TRAF1-C5*)	9	1
rs7037673 (*TRAF1-C5*)	9	1
rs10760130 (*TRAF1-C5*)	9	1
rs10985073 (*TRAF1-C5*)	9	1

From Table 1, each two-locus set includes rs2476601 (actually, all 25 retained two-locus sets include rs2476601). Although rs2476601 (*PTPN22*) is also top-ranked when only considering marginal effect (Table 2), it affects the trait variation even more (with a much larger *T *value) when considered jointly with another SNP. Three SNPs in *TRAF1-C5*, rs17611, rs993247, and rs1951784, are not significant in single-locus sets, but stand out when interacting with rs2476601 in two-locus sets. rs7037673 (*TRAF1-C5*) is as active as rs3761847 (*TRAF1-C5*) in both single- and two-locus sets. However, we did not get any evidence supporting *rs7574865 *(*STAT4*).

In summary, considering three genes together, *TRAF1-C5 *and *PTPN22 *interact significantly, with the best two-locus set being rs7037673 and rs2476601. In the gene *TRAF1-C5*, we discovered SNP rs7037673, which is as active as rs3761847 in both single-locus and two-locus sets. However, we did not detect any significant role for *rs7574865 *(*STAT4*).

## Discussion

The primary advantage of MCSM is that it utilizes multiple traits at the same time to detect gene-gene interactions. However, the computations become intensive due to the trait matrix instead of a trait vector, so we only considered one-locus and two-locus sets in our current analysis. To reduce the computational burden, certain efficient screening statistic or any machine-learning methods could be used.

The other concern is the validity of our simulated phenotypes in controls. From the statistical point of view, we could impute them by empirical parameters, and our method works well as long as the traits' distributions in cases and controls largely differ from each other. However, whether such imputated phenotypes are reasonable and meaningful in the biological view still needs further discussion.

## List of abbreviations used

AIC: Akaike's information criterion; Anti-CCP: Anti-cyclic citrullinated peptide; GAW16: Genetic Analysis Workshop 16; IgM: Immunoglobulin M; MAF: Minor allele frequency; MCSM: Multivariate combinatorial searching method; RA: Rheumatoid arthritis; SNP(s): Single-nucleotide polymorphism

## Competing interests

The authors declare that they have no competing interests.

## Authors' contributions

XC carried out the data cleaning, evaluated the algorithm, performed the simulation and analysis, and drafted the manuscript. QS and SZ participated in the development of the algorithm. H-SC initiated and designed the study, helped develop the algorithm, guided the overall study, and helped with the manuscript. All authors read and approved the final manuscript.
